# Proceedings of the Buffalo Medical and Surgical Association

**Published:** 1888-11

**Authors:** Wm. A. Hoddick

**Affiliations:** Secretary pro tem.


### *PROCEEDINGS OF THE BUFFALO MEDICAL AND SURGICAL ASSOCIATION.*

Reported by WM. A. HODDICK, M. D., Secretary *pro tem.*

Regular monthly meeting of the Buffalo Medical and Surgical Association, held October 2, 1888, at 8.30 P. M., in the Mechanics' Institute Building, the President, Dr. P. W. VAN PEYMA, in the chair. Present: Drs. Bartlett, Norton, Wetmore, Potter (W. W.), Falk, Howell, Potter (F. H.), Park, Wende, Stockton, Bissell, Brecht, Dorland, Snow, Hayd, Hartwig, Hebenstreit, Pryor, Skinner and Flagg.

The minutes of the last meeting were read and approved.

DR. J. D. HILL was elected a member of the Association.

DR. HARTWIG read a paper on "Chronic Pneumonia."

DR. PRYOR followed with an interesting paper on "Treatment by the Pneumatic Cabinet."

In the discussion of the papers which followed, DR. BARTLETT said that he had treated several cases of phthisis during the past year, in which tubercular bacilli had been demonstrated by microscopic examination, and yet the disease terminated favorably in cure under treatment. He thought the cabinet useful in incipient cases, and urged greater care in diagnosis in the earlier stages of the disease. He thought that specialists, and laryngologists in particular, should include in their studies also diseases of the deeper structures, lungs, bronchi, etc. The present mode of treatment seemed to be drifting toward antisepsis. Fumes of sulphur had in many of his cases proved beneficial, although in some they irritated the mucous membrane.

DR. F. H. POTTER emphasized the statement made in Dr. Pryor's paper in regard to the cough being due to irritation in the pharynx and upper air passages. He recommended the use of Semple's inhaler. He had used menthol in solutions varying from thirty to fifty per cent. in strength, with good results.

DR. PARK wished to know if the air-pressure gauge used in the cabinet was equivalent to pressure as measured by the barometer. He thought that matter of atmospheric pressure did not receive sufficient consideration, and that in the selection of climate one with an equitable atmospheric pressure be advised.

Dr. Bartlett had patients in California who had been greatly benefited by the change of climate.

Dr. Hartwig asked if the pressure could be varied during inspiration and expiration.

Dr. Pryor, in closing the discussion, premised by saying that he did not believe in the existence of chronic pneumonia. In the incipient stages of pulmonary disease, the mechanical effect of the cabinet was to aid in stretching up indolent and indurated portions of the lung. He had not experimented with oxygen. Had used menthol with benefit. Cough is generally due to a pharyngitis, not to irritation in the lungs, and disappears under appropriate local treatment. He agreed with Dr. Park that insufficient attention was given to atmospheric pressure. The pressure as measured in the cabinet was equivalent to pressure measured by the barometer. He generally used from one-half to one pound. The pressure could be brought to bear either on the surface of the body or in the lung, causing forcible inspiration or expiration. In regard to sending patients out of town—if they could afford it, well and good, but he thought it wrong to send them away during the later stages, after septic symptoms have developed.

Dr. Lothrop was given further time in which to prepare a memorial on the death of the late Dr. Campbell.

Small-pox was given as the prevailing disease. In its discussion, Dr. Bartlett said that he had little faith in bovine virus, and that his experience during the past month intensified his distrust as to the quality and quantity of virus on the points. He suggested the formation of a syndicate to establish a plant for the propagation of virus, and to control its sale to physicians.

Dr. Wende mentioned the fact of his brother, the veterinary surgeon, having several cases of cow-pox under treatment. He had encouraged him to inoculate some calves, and invited physicians interested to call upon him.

Dr. Hayd attributed the better results he had obtained of late to the dulling of his instrument. He thought six spots should be made, and cited statistics to show a decrease in mortality proportionate to the number of inoculations.

Dr. Hartwig had an abscess develop on an arm which he had vaccinated.

Dr. Dorland added his testimony as to the efficiency of vaccination in preventing small-pox, and also its communicability during its early stages, by citing a case where a family of six had been exposed during the first stages of the disease, and then vaccinated. Five of the inoculations were successful, but the sixth, which did not take, was subsequently taken down with small-pox.

Dr. Hartwig believed vaccination after exposure to be a prophylactic.

Essayists for the next meeting were announced: Dr. W. A. Wheeler, subject, "Certain Diseases of the Testicle;" and Dr. H. R. Hopkins, subject, "Basedow's Disease."

Adjourned till Thursday, November 8th.

